# Bridging the Regulatory Chasm in Investigator‐Initiated Human Subject Cannabis Research

**DOI:** 10.1002/cpt.70195

**Published:** 2026-01-07

**Authors:** Heather M. Barkholtz, William Naviaux

**Affiliations:** ^1^ Pharmaceutical Sciences, School of Pharmacy University of Wisconsin‐Madison Madison Wisconsin USA; ^2^ Forensic Toxicology Section, Environmental Health Division Wisconsin State Laboratory of Hygiene Madison Wisconsin USA

## Abstract

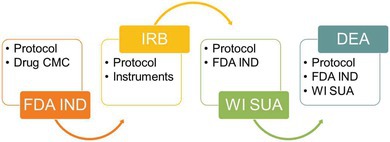

Despite widespread cannabis use and increasing public health concerns, federally compliant human pharmacology studies on cannabis remain exceedingly rare. This perspective details the complex and duplicative regulatory pathways that constrain rigorous investigation. Building on federal reports and calls to action, systemic reform is essential to support the rigorous human research needed to inform evidence‐based cannabis policy.

Despite rising public interest and expanding legalization, rigorous clinical research on cannabis and its constituents remains alarmingly limited in the United States.^1^ While thousands of publications reference cannabis, studies focused on clinical trials, pharmacology, and impairment remain limited compared to other widely used unscheduled psychoactive substances, as shown in **Figure** **1**. This scarcity of high‐quality evidence is not due to lack of scientific interest or public health need but stems instead from a convoluted and duplicative regulatory framework that disincentivizes researchers from pursuing federally compliant studies, a reality that persists despite recent federal actions directing the U.S. Drug Enforcement Administration (DEA) to initiate rescheduling of cannabis from Schedule I to III. Drawing from our experience conducting a pilot clinical study comparing the pharmacology of delta‐9‐tetrahydrocannabinol (Δ^9^‐THC) and delta‐8‐tetrahydrocannabinol (Δ^8^‐THC), this perspective outlines the logistical complexities that impede human subjects’ cannabis research and offers context for the enduring evidence gap.

**Figure 1:**
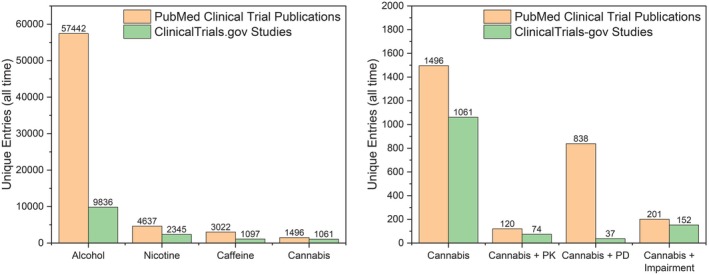
Comparison of widely available psychoactive substance‐related publication volume and registered clinical studies across bibliographic and trial registries. PubMed and ClinicalTrials.gov were queried on 17 December 2025, using the search terms indicated on the x‐axis. PubMed searches were restricted to the *Clinical Trial* article type filter. For PubMed queries containing two terms, the operator “AND” was applied (e.g., *cannabis AND pharmacokinetics*). ClinicalTrials.gov searches were conducted using the first specified x‐axis term as the *intervention* and second term as *Other Terms*, with no additional filters. For clarity, pharmacokinetics is abbreviated PK and pharmacodynamics as PD although the full word was used to query registries. The figure illustrates the relative scarcity of registered interventional studies and controlled clinical pharmacology investigations involving widely available psychoactive substances, highlighting a persistent gap between public accessibility and rigorously designed human research.

## THE INVESTIGATIONAL NEW DRUG APPLICATION: FDA’S ROLE

All clinical research involving administration of cannabis or cannabinoids must begin with an Investigational New Drug (IND) application submitted to the U.S. Food and Drug Administration (FDA). Despite widespread cannabis and cannabinoid product availability in both regulated and unregulated markets, they are considered drugs as defined in section 201(g)(1) of the FD&C Act (21 U.S.C. 321(g)(1)), requiring FDA oversight for administration in clinical investigations. Cannabis research may be conducted under the FDA’s guidance for botanical drug development which accommodates the unique nature of plant‐based medicines while maintaining the same high standards of safety and efficacy as any conventional single‐molecule drug.^2^ Although exemptions from an IND are possible, they are limited to products lawfully marketed in the United States and protocols that do not increase the risk through novel dosing, routes of administration, or vulnerable populations.^3^ Products lawfully marketed in the United States include Epidiolex (purified CBD derived from cannabis), Marinol and Syndros (synthetic Δ^9^‐THC), and Cesamet (nabilone, a synthetic cannabinoid). Clinical trials assessing these FDA‐approved substances may not require an IND; however, this narrow scope excludes the vast array of phytocannabinoids, analogs, and formulations currently being consumed by millions of Americans. This underscores the critical need to enable rigorous scientific investigation of cannabis‐derived compounds beyond the few molecules and formulations approved for therapeutic use.

Preparing an IND demands a level of expertise, infrastructure, and financial investment typically available only to pharmaceutical companies during drug development. Investigators must provide important yet costly to produce data demonstrating drug product quality and consistency. This includes chemistry, manufacturing, and control (CMC) information about the cannabinoid formulation, pharmacology and toxicology data—often unavailable—and a fully designed clinical protocol.^2^ Pharmacology and toxicology literature for emerging semi‐synthetic cannabinoids like Δ^8^‐THC, Δ^10^‐THC, hexahydrocannabinol (HHC), etc. is sparse^4^ necessitating creative justification or investment in gathering preclinical animal data to satisfy FDA reviews. Meanwhile, these products proliferate the commercial marketspace and are consumed en masse and without safety data by the American population.^5^ This underscores the disconnect between regulatory oversight for clinical research and the realities of widespread, unregulated use.

Despite the fact that 40 US states now allow “medical” cannabis use, researchers must use cannabinoid products manufactured in compliance with good manufacturing practices (GMP) to ensure purity, potency, and stability^2^ Since cannabis remains illegal at the federal level, the FDA does not currently mandate GMP for state‐legal cannabis dispensaries and manufacturers. Put another way, current federal cannabis regulation prevents investigators from studying cannabis and cannabinoid products in forms and from sources most representative of real‐world use. GMP requirements, while well intended, often push the cost of producing and characterizing clinical‐grade cannabinoid products into the six‐figure range, creating a major financial barrier for investigator‐initiated studies. Although the NIH recently eliminated the requirement for investigators requestiong $500,000 or more in direct costs in any budget year to obtain prior approval from the Institute or Center,^6^ the practical funding constraints facing cannabis researchers remain largely unchanged. The nominal $500,000 direct cost benchmark for most R01 applications has not increased in decades, despite rising regulatory, manufacturing, and compliance costs. Moreover, under the NIH’s peer review framework, study feasibility carries substantial weight in funding decisions; however, demonstrating feasibility is particularly challenging for cannabis studies. To meet FDA requirements, investigators must first purchase or produce the cannabis/cannabinoid product to generate the CMC data necessary for an IND submission, a process requiring considerable financial investment well before a competitive NIH grant application can be submitted. This dynamic creates a situation where regulatory‐driven price inflation collides with stagnant or decreasing federal funding, making rigorous clinical research on cannabinoids financially unattainable for many investigators.

## INSTITUTIONAL REVIEW BOARDS AND HUMAN SUBJECTS OVERSIGHT

Concurrent with IND submission, researchers must obtain Institutional Review Board (IRB) approval. This step, while standard in human subjects’ research, increases in complexity when dealing with federally controlled substances. Schedule I substances are defined as having high abuse potential, no accepted medical use (despite FDA approval and DEA rescheduling of Marinol, Syndros, and Cesamet), and lack of accepted safety even under medical supervision. These criteria can make IRBs especially cautious, often requiring extensive justification and safeguards before approving research protocols.

## STATE‐LEVEL OVERSIGHT: THE CASE OF WISCONSIN

Following FDA and IRB clearance, researchers must navigate state‐level controlled substances regulatory hurdles. In Wisconsin, for example, cannabis and all THC isomers remain Schedule I substances under state law. To legally purchase, possess, and administer cannabis or psychoactive cannabinoids in human research, investigators must obtain a Special Use Authorization (SUA) through the Wisconsin Controlled Substances Board. The SUA application process is detailed and time‐consuming, requiring submission of several forms, a protocol summary, investigator credentials, secure drug handling plans, and justifications for each Scheduled substance. Approval can take months, and any protocol amendments or product formulation changes require reapproval. These delays pose significant logistical barriers, particularly for pilot studies funded on limited timelines and budgets. Moreover, because the SUA is investigator‐ and site‐specific, multicenter trials involving cannabis may require separate authorizations at each site. This localized regulatory heterogeneity presents yet another deterrent to expanding the clinical evidence base.

## DEA RESEARCHER LICENSURE: THE FINAL GATEKEEPER

To legally purchase cannabis or psychoactive cannabinoids and administer them to humans, researchers must secure a Schedule I researcher license from the DEA, except in the rare cases of FDA‐approved cannabinoid drugs that have been rescheduled.^3^ Although the DEA has been directed to initiate rulemaking to reschedule cannabis to Schedule III, this process has not yet eliminated or materially reduced the regulatory requirements governing cannabis research. This can only be accomplished after FDA IND and IRB approvals and securing any local controlled substances licensure. DEA researcher applications include forms, the full protocol, FDA IND “May Proceed” letter, IRB approval letter, copies of local controlled substance licensure, investigator credentials, and secure drug handling plans. Applications are reviewed by the DEA in collaboration with the Department of Health and Human Services (HHS), namely the FDA—despite the FDA IND review process. The DEA’s review involves facility inspections, security assessments, and study team member background checks. Even experienced research institutions may lack the infrastructure for Schedule I research, requiring installation of compliant safes, surveillance, and tracking systems.

Recently, the US HHS conducted a scientific review of cannabis and reported a growing body of evidence suggesting accepted medical uses and a lower potential for abuse compared to other Schedule I substances.^7^ This review informed a recommendation to reschedule cannabis to Schedule III, and the DEA has been directed to initiate this process. However, as of this writing, cannabis and its primary psychoactive constituents ‐ including Δ^9^‐THC ‐ remain classified as Schedule I under federal law, meaning every stage of production, formulation, distribution, and possession for research requires specific DEA licensure. As of this writing, only a limited number of DEA‐registered manufacturers are authorized to supply cannabis for research.^8^ This includes the National Institute on Drug Abuse (NIDA) Drug Supply Program and a handful of private companies under new licensing policies. Accessing diverse cannabinoid formulations, reflective of the commercial cannabinoid market, remains a substantial hurdle. This framework sharply contrasts with the commercial availability and mass consumption of cannabinoid products in the United States, a reality that federal oversight has not kept pace with.^5^

## THE RIPPLE EFFECT ON CLINICAL EVIDENCE

Interlocking regulatory requirements: the FDA IND, IRB approval, state‐level schedule I drug authorization, and DEA registration impose a multilayered regulatory burden that makes both initial approval and continued compliance prohibitively challenging for many investigators. The process demands significant time, funding, and institutional support with little flexibility. Consequently, much of the cannabinoid data informing clinical decision making today comes from observational studies, surveys, or trials conducted in jurisdictions with more permissive regulatory environments.^9^ For example, several U.S. states that have legalized adult‐use cannabis permit “Green Lab” training programs modeled after traditional alcohol “wet labs” to educate law enforcement officers on cannabis impairment detection.^10^ In these programs, volunteers bring and consume their own legally obtained cannabis prior to structured interactions with law enforcement trainees. Although these exercises provide valuable experimental insights that inform law enforcement and traffic safety practices, they necessarily rely on heterogeneous, non‐standardized cannabinoid products and therefore lack the rigor of controlled clinical trials. Their results are also often missing from the peer‐reviewed scientific literature, leaving a critical gap between applied field observations and formal clinical pharmacology research.

This fragmented approach to cannabis research not only delays our understanding of therapeutic efficacy and safety but also impedes policy development and patient care. As public access to cannabis widely outpaces scientific consensus, there is a growing imperative to streamline the federal framework and incentivize investigator‐initiated research.

## MOVING FORWARD

Policymakers and regulatory agencies must consider pathways that retain essential safety oversight while reducing redundant barriers to research. While DEA rescheduling to Schedule III may alleviate certain administrative and tax‐related constraints, it does not, on its own, resolve the FDA, IRB, state‐level, and manufacturing barriers that uniquely burden investigator‐initiated cannabis research. For example, harmonizing DEA and state licensing requirements, reconsidering cannabis scheduling, expanding access to research‐grade cannabis products (including isolates of naturally occurring and semi‐synthetic THC‐isomers currently not included in the NIDA Drug Supply Program), permitting the use of commercially available cannabis products authorized for sale under state regulatory frameworks, and modernizing the IND process for cannabinoids could dramatically lower the entry threshold for conducting meaningful human research. Until such reforms are implemented, a persistent gap will remain between widespread cannabis use and rigorously designed human research, reflecting the relative scarcity of controlled clinical studies needed to understand its health effects.

## FUNDING

Funding for this project was provided by NIH/NCATS through CTSA award UL1TR002373 to the UW Institute for Clinical and Translational Research. HB received mentored professional development through the University of Wisconsin‐Madison STRIDE program. Funding for STRIDE was provided by the UW School of Medicine and Public Health from the Wisconsin Partnership Program (Grant ID #5132) through a grant to the UW Institute for Clinical and Translational Research. The UW Institute for Clinical and Translational Research also receives funding from NIH‐NCATS Clinical and Translational Science Award (CTSA) 1UL1TR002373.

## CONFLICT OF INTEREST

The authors declared no competing interests for this work.
